# Right anterior thoracotomy vs. upper hemisternotomy for aortic valve replacement with Perceval S: is there a difference?

**DOI:** 10.3389/fcvm.2024.1369204

**Published:** 2024-10-25

**Authors:** Bogdan Okiljevic, Tatjana Raickovic, Igor Zivkovic, Petar Vukovic, Miroslav Milicic, Ivan Stojanovic, Petar Milacic, Slobodan Micovic

**Affiliations:** ^1^ Cardiac Surgeon, Clinic for Cardiac Surgery, Dedinje Cardiovascular Institute, Belgrade, Serbia; ^2^ Faculty of Medicine, University of Belgrade, Belgrade, Serbia

**Keywords:** aortic valve replacement, minimally invasive, sutureless valve, thoracotomy, hemisternotomy

## Abstract

**Background:**

Our study aimed to evaluate the early outcomes of aortic valve replacement with Perceval S sutureless valve through the right anterior thoracotomy and upper hemisternotomy approaches, and to determine if there are any differences between these two approaches.

**Methods:**

We carried out a study using data from 174 patients who underwent minimally invasive Perceval S valve implantation for aortic valve stenosis between January 2018 and August 2023. This was a retrospective, single-center observational study. The patients were divided into two groups: the hemisternotomy group (*n* = 100) and the right anterior thoracotomy group (*n* = 74).

**Results:**

The overall in-hospital mortality was 1,7%. The cardiopulmonary bypass and cross-clamp times were longer in the right anterior thoracotomy group (*p* < .001). There were no statistically significant differences in terms of stroke, paravalvular leak, mechanical ventilation time, blood transfusion requirements, pacemaker implantation, reexploration for bleeding, conversion, wound infection, or in-hospital stay. Postoperative chest drainage was lower (*p* < .001) and postoperative atrial fibrillation occurred less frequently (*p* = .044) in the right anterior thoracotomy group. The median intensive care unit stay was shorter in the right anterior thoracotomy group (*p* = .018).

**Conclusion:**

Aortic valve replacement with the Perceval S valve through either an upper hemisternotomy or a right anterior thoracotomy is a procedure associated with low perioperative complication rates. Right anterior thoracotomy for an aortic valve replacement with the Perceval S valve was associated with lower postoperative bleeding, a lower postoperative atrial fibrillation incidence and a shorter intensive care unit stay compared to upper hemistornotomy.

## Introduction

Aortic valve stenosis is the most common valve lesion that requires surgical or transcatheter treatment in Europe and North America (1). Despite advances in transcatheter aortic valve replacement, the surgical treatment of aortic stenosis still represents one of the most common cardiac surgical procedures and implies aortic valve replacement (AVR) with a mechanical or biological valve. Conventional AVR (cAVR) is performed through a full median sternotomy approach. Minimally invasive approaches are increasingly used in heart surgeries. Minimally invasive aortic valve replacement (miAVR) is an alternative to cAVR. According to the American Heart Association, minimally invasive cardiac surgery is a procedure that uses “a small chest wall incision that does not include the conventional full sternotomy” (2).

miAVR was described and popularized in the 1990s and has become a standard in most high-volume centers. miAVR is associated with less bleeding, a lower transfusion rate, a shorter time of mechanical ventilation, a shorter intensive care unit (ICU) stay and a shorter in-hospital stay compared to cAVR. These results are described in many clinical studies, despite the increased technical complexity of the miAVR procedure, which leads to increased operative, cardiopulmonary bypass, and cross-clamp times (3–6). With the introduction and widespread application of rapid deployment valves, which facilitates the valve implantation procedure, miAVR cardiopulmonary bypass and cross-clamp times were reduced by approximately 40% (7). These reductions in operative times led to better postoperative results and decreased mortality (8).

Today, the most commonly used miAVR approaches are upper hemi-sternotomy (HS) and right anterior thoracotomy (RAT). Few studies are comparing the HS and RAT approach for aortic valve replacement, which have shown similar in-hospital mortality, blood transfusion, and stroke rates. These studies demonstrated that HS is slightly better than RAT in terms of reoperation for bleeding, aortic cross-clamp time, and conversion to sternotomy, while RAT is slightly better than HS in terms of the length of postoperative mechanical ventilation and length of hospital stay (9, 10).

The Perceval S aortic valve bioprosthesis (Corcym, Italy) is a representative and one of the most commonly used rapid deployment valves. The Perceval valve was developed to combine the advantages of transcatheter aortic valve replacement (allowing a fast implantation) and surgical aortic valve replacement (allowing for removing native aortic valve and meticulous decalcification of aortic annulus) (11). Besides easy and fast valve implantation through minimally invasive approaches, the Perceval aortic valve has shown favorable clinical and hemodynamic results at mid-term, with freedom from valve-related major adverse events at 5 years of 96,93% (12).

This study aims to analyze the data from our institution to provide a comparison of the outcomes between the HS and RAT approaches for surgical aortic valve replacement with Perceval S sutureless valve in patients with isolated aortic stenosis.

## Material and methods

### Patient selection and data collection

A group of authors from a high-volume institution that perform around 450 SAVR each year conducted a retrospective analysis of 174 patients who underwent miAVR Perceval valve implantation for isolated aortic valve stenosis between January 2018 and August 2023. Our center has 10 surgeons who do upper hemisternotomies, and five of them perform a right anterior thoracotomy for SAVR. Relatively small number of implanted Perceval prothesis in this period is due to limited budget of health care system in our country.

The local Ethical Committee approved data collection for this study, and patient consent was waived due to the retrospective nature of the study. Inclusion and exclusion criteria are shown in Table 1.

**Table 1:** Inclusion and exclusion criteria.

Inclusion criteria	Exclusion criteria
Age ≥ 18 years	Acute endocarditis
Echocardiographic diagnosis of severe aortic valve stenosis	Previous cardiac surgery
Life expectancy ≥1 year	Bicuspid aortic valve with asymmetric sinus of Valsalva
Perceval valve implantation	Dilatation of ascending aorta ≥4 cm
RAT or HS approach	Ratio between the diameter of sinus of Valsalva and diameter of aortic anulus above 1,3
	Preoperative hemodynamic instability or mechanical circulatory support
	Deformation of thorax
	Current systemic infection
	Need for urgent operation
	Requirement of concomitant coronary artery bypass grapht surgery, ascending aorta or other valve surgery

Patient demographics, preoperative, operative, and postoperative data were collected from the electronic cardiac surgery department database. The final sample contained data for 174 patients, divided into two groups depending on the procedural approach: HS-AVR and RAT-AVR.

### Preoperative planning and surgical procedure

All patients scheduled for the miAVR underwent detailed preoperative transthoracic echocardiography, performed by an experienced sonographer. Accurate assessment of the aortic valve, aortic annulus, sinuses of Valsalva, and ascending aorta was performed. All miAVR procedures were performed by experienced cardiac surgeons. The approach was determined by the surgeon's preference. Patients selected for the RAT approach underwent multislice computed tomography without and with contrast to evaluate the anatomic relationship of the aortic valve, ascending aorta, sternum, and intercostal spaces. As proposed by Glauber (13), patients were suitable for the RAT approach if, at the level of the main pulmonary artery, more than half of the ascending aorta was to the right with respect to a vertical line drawn from the right sternal border, and the distance from the ascending aorta to the sternum did not exceed 10 cm. If the patient did not fulfill those criteria, the operation was done through the HS approach.

Patients with bicuspid aortic valve were excluded from the study.

The patients were positioned supine. After induction of the anesthesia, endotracheal intubation with a single-lumen endotracheal tube for HS-AVR and a double-lumen endotracheal tube for RAT-AVR was done. External defibrillator pads were placed on the chest wall.

For the HS-AVR approach, a 6–8 cm vertical midline skin incision was made, starting above the manubriosternal angle to the third intercostal space level. Upper J hemisternotomy in the third or fourth intercostal space was made. Direct aortic cannulation was performed with EOPA arterial cannula (Medtronic, Minneapolis, Minn) and venous canulation was performed mostly through the superior caval vein in the right atrium with 29 Fr Optiflow Venous cannula (Livanova, London, UK). In rare cases, a standard double-stage atriocaval cannula was placed directly through the right atrium or percutaneous venous cannulation through femoral vein was performed.

For RAT-AVR, a 5–7 cm horizontal skin incision over the anterior part of the right second intercostal space was made. After thoracotomy, the right internal mammary arterty and vein are ligated and divided. After assessment of the aortic root position, the second or third rib was detached from the sternum. A soft tissue retractor was placed. Direct aortic cannulation was performed with EOPA arterial cannula (Medtronic, Minneapolis, Minn) and venous canulation was performed mostly through the superior caval vein in the right atrium with 29 Fr Optiflow Venous cannula (Livanova, London, UK). In rare cases, the cut-down femoral artery and vein cannulation was performed.

After the establishment of the cardiopulmonary bypass, a left heart vent was placed through the upper right pulmonary vein, and patient was cooled down to mild hypothermia. Standard DeBakey aortic clamp or Glauber clamp (Cardiomedical GmbH, Germany) was used for the aortic cross-clamping. Antegrade Del Nido cardioplegia was given into the aortic root or selectively into the coronary ostia after aortotomy.

The Perceval S valve implantation was done in accordance with recomendations (14). Predominantly we used this prothesis for the patients with small aortic anulus, less than 21 mm.

Transversal aortotomy was made 2 cm higher than ordinary. After the aortic valve excision, annulus decalcification, and appropriate sizing, three guiding sutures were placed at the nadir point of each sinus of Valsalva. The Perceval valve was deployed using the dedicated delivery system and balloon post-dilatation was performed. After careful inspection of the valve and the aortic root, the aortotomy was closed in the usual manner.

A temporary ventricular pacemaker wire was placed on the diaphragm surface of the right ventricle, deaeration maneuvers were performed and the aortic cross-clamp was removed. Control transesophageal echocardiography was routinely performed to check the function, hemodynamics, and position of the valve and to exclude significant paravalvular leakage. If the paravalvular leak was graded as more than mild, the aorta was reclamped and the Perceval valve was retrieved and redeployed.

Pericardial and pleural (if pleural space was opened) drains were inserted and closure of the chest was done in usual manner. Patients were transported sedated and intubated in the ICU.

### Study outcomes

The endpoints of this study were cardiopulmonary bypass and cross-clamp times, more than mild paravalvular regurgitation, valve redeployment, mechanical ventilation time, postoperative drainage, reexploration for bleeding, conversion to another surgical approach, ICU and hospital stay, transfusion of packed red blood cells, heart block that requires permanent pacemaker implantation, new-onset postoperative atrial fibrillation, postoperative renal failure that requires dialysis, stroke and in-hospital mortality.

### Statistical analysis

Continuous data are presented as the mean ± standard deviation or median and interquartile range (IR). Categorical data are presented as numbers (percentages). Descriptive statistics measures were used: arithmetic mean, standard deviation, median, quartiles, frequencies, and percentages. For numeric variables, the *t*-test for independent samples and the Mann–Whitney test were used to detect differences between the groups. A *p* < 0.05 value was taken for the statistical significance of the test. Statistical analysis was performed using IBM SPSS Statistics for Windows, Version 25.0 (IBM Corp., Armonk, NY, USA).

## Results

A total of 174 patients underwent miAVR with the Perceval S sutureless valve during the study period. Of these, 100 patients (57,5%) underwent HS-AVR and 74 (42,5%) underwent RAT-AVR. The baseline characteristics of the two groups are shown in Table 2. Patients in the HS-AVR group were older (73.2 ± 5.2 vs. 70.7 ± 5.7; *p* = .004) and had more often the diagnosis of extracardiac arteriopathy (28% vs. 13,5%, *p* = .022) compared with patients in RAT-AVR group. There was no significant difference between both groups regarding the other preoperative patient characteristics.

**Table 2:** Baseline characteristics.

Characteristics	HS-AVR (*n* = 100)	RAT-AVR (*n* = 74)	*p* value
Age (y)	73.2 ± 5.2	70.7 ± 5.7	.004^a^
Female gender	67 (67%)	51 (68.9%)	.789^b^
Body mass index	27.7 ± 4.2	27.5 ± 4.8	.769^a^
Body surface area	1.9 ± 0.2	1.9 ± 0.2	.368^a^
LVEF (%)	53.9 ± 8.9	54.8 ± 8.1	.495^a^
LVEF < 30%	4 (4%)	1 (1.4%)	.396^c^
Euroscore II	1.9 (2.1)	1.5 (1.5)	.160^d^
Diabetes mellitus	38 (38%)	22 (29.7%)	.256^b^
Hyperlipidemia	70 (70%)	53 (71.6%)	.816^b^
COPD	8 (8%)	5 (6.8%)	.758^b^
Hypertension	93 (93%)	66 (89.2%)	.376^b^
Smoking	41 (41%)	30 (40.5%)	.951^b^
History of CVI	6 (6%)	4 (5.4%)	1.00°^c^
NYHA class III-IV	50 (50%)	29 (39.2%)	.157^b^
Exracardiac arteriopaty	28 (28%)	10 (13.5%)	.022^b^
CKD	14 (14%)	5 (6.8%)	.130^b^
Pulmonary hypertension	58 (58%)	39 (52.7%)	.487^b^

Data presented as mean ± standard deviation, *n* (%) or median (interquartule range).

CKD, chronic kidney disease; COPD, chronic obstructive pulmonary disease; CVI, cerebrovascular insult; HS-AVR, hemisternotomy aortic valve replacement; LVEF, left ventricle ejection fraction; NYHA, New York Heart Association; RAT-AVR, right anterior thoracotomy aortic valve replacement.

^a^ Independent samples *t* test.

^b^ Peason chi square test.

^c^ Fisher's exact test.

^d^ Mann-Whitney *U* test.

Intraoperative characteristics are summarized in Table 3. Patients in the HS-AVR group had a shorter cross-clamp (46.6 ± 13.5 vs. 55.1 ± 14.4; *p* < .001) and a cardiopulmonary bypass times [71.5 (25.5) vs. 83 (28); *p* < .001] compared with patients in RAT-AVR group. There were three patients (one in HS-AVR and two in the RAT-AVR group) with an intraoperative diagnosis of paravalvular leakage that required valve redeployment (no one required a switch to another aortic valve prosthesis). One patient from the HS-AVR group required conversion to sternotomy due to aortic root bleeding. Two patients from the RAT-AVR group required conversion, one to sternotomy due to paravalvular leakage and Perceval valve redeployment and the second to hemisternotomy due to unfavorable anatomy. We had two reoperations due to valve problems (both in the HS-AVR group), first due to an early prosthetic infective endocarditis and second due to a paravalvular regurgitation that was underestimated on intraoperative transesophageal ultrasound exam. The most common Perceval valve size used in the HS-AVR group was M (34%) and in the RAT-AVR group S (36,5%). The least commonly used size in both groups was XL (15% in the HS-AVR group and 8,1% in the RAT-AVR group).

**Table 3:** Intraoperative characteristics.

Characteristics	HS-AVR	RAT-AVR	*p* value
CPB time (min)	71.5 (25.5)	83 (28)	<.001^a^
Cross-clamp time (min)	46.6 ± 13.5	55.1 ± 14.4	<.001^b^
PVL > 1	1 (1%)	2 (2.7%)	.576^c^
Intravalvular leak > 1	0 (0%)	0 (0%)	/
Valve redeployement	1 (1%)	2 (2.7%)	.576^c^
Conversion to other approach	1 (1%)	2 (2,7%)	.576^c^
Perceval S size			
S	23 (23%)	27 (36.5%)	.052^d^
M	34 (34%)	21 (28.4%)	.430^d^
L	27 (27%)	20 (27%)	.997^d^
XL	15 (15%)	6 (8.1%)	.168^d^

Data presented as mean ± standard deviation, *n* (%) or median (interquartule range).

CPB, cardiopulmonary bypass; HS-AVR, hemisternotomy aortic valve replacement; PVL, paravalvular leak; RAT-AVR, right anterior thoracotomy aortic valve replacement.

^a^ Mann-Whitney *U* test.

^b^ Independend samples *t* test.

^c^ Fisher's exact test.

^d^ Pearson chi square test.

Overall, in-hospital mortality was 1,7%, with no significant difference between the two groups (2% in the HS-AVR group and 1,4% in the RAT-AVR group), Table 4. The reasons for fatal outcome in the HS-AVR group were postoperative respiratory failure and systemic inflammatory response syndrome, while in the RAT-AVR group it was acute renal failure. Patients undergoing Perceval S implantation through the RAT-AVR approach had a lower chest tube drainage [200 (100) ml vs. 300 (200) ml; *p* = .0007] and a lower incidence of postoperative atrial fibrillation (17,6% vs. 31%, *p* = .044). There were no significant differences in stroke, reexploration for bleeding, mechanical ventilation time, pacemaker implantation, blood transfusion rate, reoperation due to valve problem, incidence of wound infection, and hospital stay. Patients in the RAT-AVR group had shorter ICU stays [median, 1,5 (2) vs. 2 (2) days; *p* = .018].

**Table 4:** Postoperative outcomes.

Characteristics	HS-AVR	RAT-AVR	*p* value
Mortality	2 (2%)	1 (1.4%)	1.000^a^
Stroke	1 (1%)	2 (2.7%)	.576^a^
Reexploration for bleeding	5 (5%)	3 (4.1%)	1.000^a^
Mechanical ventilation time (hr)	12.5 (6)	14 (7)	.068^b^
Chest tube output (ml)	300 (200)	200 (100)	.006^b^
Pacemaker implantation	2 (2%)	5 (6.8%)	.137^a^
Blood transfusion	51 (51%)	36 (48.6%)	.759^c^
Blood transfusion (units)	1 (1)	1 (1)	.444^a^
Postoperative atrial fibrilation	31 (31%)	13 (17.6%)	.044^c^
Reoperation due to valve problem	2 (2%)	0 (0%)	.508^a^
Wound infection	2 (2%)	1 (1.4%)	1.000^a^
ICU stay (days)	2 (2)	1.5 (2)	.018^a^
Hospital stay (days)	7 (3)	7 (4)	.802^a^

Data presented as mean ± standard deviation, *n* (%) or median (interquartule range).

HS-AVR, hemisternotomy aortic valve replacement; ICU, Intensive Care Unit; RAT-AVR, right anterior thoracotomy aortic valve replacement.

^a^ Fisher's exact test.

^b^ Mann-Whitney *U* test.

^c^ Pearson chi square test.

## Discussion

Our study demonstrates that aortic valve replacement with the Perceval S valve can be safely performed using minimally invasive approaches such as HS and RAT. These approaches are associated with low rates of perioperative morbidity and mortality.

miAVR has proven its safety over the years, with the benefit of patient satisfaction and enhanced recovery. The benefits of the miAVR have been well-documented, but data collected from most of the studies are focused on upper ministernotomy. Ministernotomy for AVR showed numerous benefits over the cAVR: less time on the ventilator, shorter ICU and in-hospital stay, decreased postoperative pain, and lower transfusion requirement (15). Similar advantages have been observed with the RAT-AVR approach compared to cAVR (3). However, it is important to note that miAVR is technically more demanding procedure due to reduced operative space with modest exposure, resulting in longer operative, CPB, and cross-clamp times, which may increase the risk of postoperative adverse events (16). The use of the sutureless valves has been shown to reduce cross-clamp and CPB times in both the most commonly used miAVR approaches – HS-AVR (17) and RAT-AVR (18).

We have observed that 45,4% of patients were in NYHA III or IV class. This is probably due to a delay in surgical intervention during the COVID-19 pandemic, so many patients on admission were highly symptomatic.

Our results indicate that AVR with the Perceval S valve through the RAT approach is a more time-consuming procedure, with longer CPB and cross-clamp times compared to the HS approach. This finding is consistent with previous studies that have reported slightly increased CPB and cross-clamp time in the RAT-AVR group compared to the HS-AVR group, although the difference did not reach statistical significance (7). These findings suggest that the RAT approach may be technically a more demanding procedure. However, in our study, there were no differences between the groups in terms of conversion to another surgical approach, paravalvular leakage, or the need for reclamping of the aorta and redeployment of the valve prosthesis. Prolonged CPB and cross-clamp times did not translate to worse clinical outcomes in our study.

We also found that patients undergoing RAT-AVR had a lower incidence of postoperative atrial fibrillation. New-onset atrial fibrillation after AVR is associated with increased in-hospital mortality and a higher risk of stroke and mid-term mortality (19). The RAT approach probably leads to less manipulation of the heart and pericardium, which may explain the lower incidence of new-onset postoperative atrial fibrilation (20).

miAVR has theoretical advantage in the reduction of bleeding compared to cAVR (21), due to limited trauma to bone structures, less dissection, and smaller pericardial incision. In most of the studies, the effect of miAVR on reduced blood loss remains unclear (9, 10). In our study, patients in the RAT-AVR group had less postoperative drainage compared to those in the HS-AVR group. However, these results did not translate to a reduction in blood transfusion rates or the amount of blood units used per patient.

A meta-analysis published by Yousuf Salmasi suggested that the increased tehnical complexity of the RAT approach for AVR may lead to a higher incidence of bleeding complications compared to the HS for AVR (9). We found no difference between the groups in terms of reoperation for bleeding. The use of the Perceval S valve simplifies the AVR procedure, which may contribute to decreased bleeding complications, especially in the RAT-AVR group.

Patients in the RAT-AVR group also had a shorter Intensive Care Unit stay. However, the shorter ICU stay did not shorten the overall hospitalization time in the RAT-AVR group. These findings may be attributed to the short hospitalization time and extensive experience of our surgical team with the HS approach for AVR.

In terms of other postoperative outcomes and complications, both access routes showed similar results. There were no differences in terms of in-hospital mortality, stroke, permanent pacemaker implantation, reoperation for valve-related problems and wound complication.

### Study limitation

Our study had several limitations. It was a retrospective analysis of our institutional results. Approach and valve selection for AVR was left to the discretion of the surgeon. The groups were not fully comparable due to the requirement of a rightward position of the ascending aorta for RAT-AVR.

## Comment

Aortic valve replacement with the Perceval S sutureless valve through either an upper hemisternotomy or a right anterior thoracotomy is a safe and reproducible procedure associated with low perioperative complication rates. Right anterior thoracotomy for an aortic valve replacement with the Perceval S valve was associated with lower postoperative bleeding, a lower postoperative atrial fibrillation incidence and a shorter intensive care unit stay compared to upper hemistornotomy. However, further studies are needed to confirm these findings and determine the optimal approach for AVR with the Perceval S valve.

## Data Availability

The raw data supporting the conclusions of this article will be made available by the authors, without undue reservation.
